# Public Pharmacists in Council

**Published:** 1914-03-07

**Authors:** 


### Public Pharmacists in Council.

INTERESTING PAPERS AT LAST WEEK'S MEETING.

A meeting of the Public Pharmacists' and Dispensers' Association was held at St. Bride Institute, E.C., last week, Mr. Jas. Hassall France, M.P.S., in the chair. Those present included Messrs. E. Castle, F. Noad Clark, J. Cairns, A. J. Gibbons, H. Skinner, A. H. Jenkin, R. Welford, R. W. Lindsey, W. E. Kinsman, A. Howell, T. W. Eaton, E. E. Smith, T. S. Goodall, O. A. Elias, P. S. Thomas, G. Town, and G. W. Gibson (Hon. Secretary). The evening was devoted to four short papers by members: "A Pharmacist in Exile," by Mr. F. E. Bullen, M.P.S.; "Notes on Radiography," by Mr. W. E. Kinsman, M.P.S.; "The Status of the Poor-Law Pharmacist," by Mr. F. Noad Clark; and "Dispensing for the Resident Insured Staff," by Mr. G. W. Gibson, M.P.S.

Mr. Bullen, who is pharmacist at H.M. Prison, Dartmoor, described the life of a pharmacist away from the lectures and meetings of his colleagues, this condition of exile being in a way compensated for by the beautiful scenery of Devon and a country life.

Mr. W. E. Kinsman's paper was full of interest to those pharmacists who do the radiographic work for their institutions, and described not merely methods of taking radiograms, but, what is equally important, their interpretation. In the large infirmaries where the junior medical staff is constantly changing it is a decided advantage to place the apparatus in the care of a permanent officer of some technical training, like the pharmacist, and, while not encroaching on the domain of the medical man, the pharmacist can render very useful service to his institution in this matter.

Mr. F. N. Clark, in a very well-informed paper, discussed "The Status of the Poor-Law Pharmacist." A curious anomaly was mentioned, in that dispensers in dispensaries for the outdoor poor have security of tenure, and yet their colleagues in responsible posts in the large infirmaries can be dismissed by the Guardians without the Local Government Board's permission. It was urged that a pharmacist who dispenses for some 500 to 700 patients per day should be in at least as favourable a position regarding his tenure of office as, say, the steward or relieving officer, etc.

Mr. G. W. Gibson, who has done work for the Association on Insurance matters, outlined a very methodical way of dealing with the large amount of clerical work in connection with Insurance prescriptions for the staff. Several incompatibilities were mentioned in his "Odd Notes," which dealt with various dispensing problems. Perhaps the biggest share of the discussion was centred on the most suitable substitute for alcohol in iodine preparations. Now that tr. iodi is used so largely the dispensary account tends to rise, due to the cost of alcohol. Although substitutes like methylated spirit and acetylene dichloride have been suggested, it was generally agreed that the disadvantages attending their use outweighed the advantage of economy.

